# Structural and Biochemical Characterization of *Chlamydia trachomatis* DsbA Reveals a Cysteine-Rich and Weakly Oxidising Oxidoreductase

**DOI:** 10.1371/journal.pone.0168485

**Published:** 2016-12-28

**Authors:** Signe Christensen, Morten K. Grøftehauge, Karl Byriel, Wilhelmina M. Huston, Emily Furlong, Begoña Heras, Jennifer L. Martin, Róisín M. McMahon

**Affiliations:** 1 Division of Chemistry and Structural Biology, Institute for Molecular Bioscience, University of Queensland, St. Lucia, Queensland, Australia; 2 School of Life Sciences, University of Technology Sydney, Broadway, New South Wales, Australia; University of Alberta, CANADA

## Abstract

The Gram negative bacteria *Chlamydia trachomatis* is an obligate intracellular human pathogen that can cause pelvic inflammatory disease, infertility and blinding trachoma. *C*. *trachomatis* encodes a homolog of the dithiol oxidoreductase DsbA. Bacterial DsbA proteins introduce disulfide bonds to folding proteins providing structural bracing for secreted virulence factors, consequently these proteins are potential targets for antimicrobial drugs. Despite sharing functional and structural characteristics, the DsbA enzymes studied to date vary widely in their redox character. In this study we show that the truncated soluble form of the predicted membrane anchored protein *C*. *trachomatis* DsbA (CtDsbA) has oxidase activity and redox properties broadly similar to other characterized DsbA proteins. However CtDsbA is distinguished from other DsbAs by having six cysteines, including a second disulfide bond, and an unusual dipeptide sequence in its catalytic motif (Cys-Ser-Ala-Cys). We report the 2.7 Å crystal structure of CtDsbA revealing a typical DsbA fold, which is most similar to that of DsbA-II type proteins. Consistent with this, the catalytic surface of CtDsbA is negatively charged and lacks the hydrophobic groove found in EcDsbA and DsbAs from other *enterobacteriaceae*. Biochemical characterization of CtDsbA reveals it to be weakly oxidizing compared to other DsbAs and with only a mildly destabilizing active site disulfide bond. Analysis of the crystal structure suggests that this redox character is consistent with a lack of contributing factors to stabilize the active site nucleophilic thiolate relative to more oxidizing DsbA proteins.

## Introduction

Disulfide bond proteins (DSB) are thiol-disulfide oxidoreductases found in the periplasm of Gram negative bacteria which catalyse the oxidative folding of disulfide bond containing substrate proteins. The first DSB protein to be described was that of the primary oxidase DsbA from *Escherichia coli* (EcDsbA) [1]. EcDsbA is a highly oxidising protein with a redox potential of -122 mV [2] which introduces disulfide bonds into folding proteins resulting in its own active site reduction. EcDsbA is subsequently returned to its active oxidized state by interaction with an integral membrane partner protein EcDsbB. The structure of EcDsbA consists of a thioredoxin catalytic domain (containing the active site motif CPHC) with an inserted helical domain [3].

Extensive efforts over many years have yielded a structural library of over a dozen bacterial DsbA proteins. These have recently been classified into two groups (DsbA-I and DsbA-II) on the basis of structural and functional features [4]. DsbA-I and DsbA-II proteins are demarcated primarily on the basis of altered central β-sheet topology, a distinction that also approximately separates DsbA proteins from Gram negative and Gram positive bacteria.

Each DsbA group can be further subdivided into two subclasses on the basis of surface features. Type DsbA-Ia and Ib groups are relatively well represented with four and five protein members respectively. By comparison, DsbA-II proteins are less well characterized; to date only three DsbA proteins have been classified as DsbA-IIa (DsbA from *Mycobacterium tuberculosis*, *Staphylococcus aureus* and *Bacillus subtilis*) whilst DsbA-IIb is solely represented by DsbA1 from the endosymbiont *Wolbachia pipientis*. Within DsbA-Ia and DsbA-Ib, member proteins have similar redox characteristics with redox potentials falling within a 10 mV or 15 mV range, respectively [5–9]. Among the DsbA-IIa proteins redox potentials are much more varied, ranging from -131 mV [8] to -80 mV [10].

It is well documented that pathways responsible for disulfide bond formation can vary markedly amongst different bacterial species [11]. In *C*. *trachomatis*, *in silico* analysis suggests that the disulfide oxidative pathway, and to some extent the isomerase pathway, resembles the canonical DSB pathways of *E*. *coli* K12. *C*. *trachomatis* possesses a gene predicted to be a homolog of the *E*. *coli* DsbA [11] hereafter referred to as CtDsbA. Immediately upstream of *dsbA C*. *trachomatis* also encodes a homolog of *E*.*coli* DsbB. This protein is predicted to be a transmembrane protein with four transmembrane helices and two cysteine-residue containing periplasmic loops. DsbB is presumably responsible for oxidizing CtDsbA in a manner analogous to the *E*. *coli* DsbA-DsbB interaction. Notably *C*. *trachomatis* does not encode a homolog of the *E*. *coli* isomerase DsbC but has a gene with significant homology to *E*. *coli* DsbD, a membrane bound electron transporter and partner protein of *E*. *coli* DsbC. Drawing on recent extensive phylogenetic analysis of the DsbD superfamily in eubacteria [12], this gene is most likely a member of the sub-family ScsB. Finally *C*. *trachomatis* was found to contain homologs to genes coding for two periplasmic *C*. *pneumonia* proteins: DsbH and DsbJ. DsbH and DsbJ are suggested to play a role in maintaining a reducing periplasm, and have not yet been reported outside of chlamydial species [13].

Here we investigated the DsbA enzyme from *Chlamydia trachomatis*, responsible for human urogenital chlamydia infections. This infection is among the most common sexually transmitted infections worldwide with an estimated 131 million urogenital cases reported globally in 2012 by the World Health Organization [14]. A common complication of genital chlamydial infection in women is pelvic inflammatory disease, which, if untreated, can lead to infertility. Furthermore, strains of *C*. *trachomatis* can also infect the ocular mucosa where it can cause blinding trachoma [15].

In the present study we confirm that CtDsbA has oxidizing enzymatic activity and a structure similar to that of other DsbA-II type proteins that contain a second non-catalytic disulfide bond. We find that CtDsbA has a particularly weak oxidizing potential for a DsbA enzyme, which appears to stem in part from its uncommon active site dipeptide motif of two uncharged amino acids. Characterization of CtDsbA expands the DsbA structural library, provides further insight into the diversity of bacterial DsbA proteins and supports continued exploration of the potential for DsbA inhibitors with multi-species activity.

## Materials and Methods

### Protein expression and purification

The recombinant CtDsbA expressed and characterized in this study was generated using residues 34 to 238 of *C*. *trachomatis dsbA* (NCBI Gene with ID 5858475, currently annotated as DsbG). A variant form of the protein (called CtDsbA-SSS) was produced by mutating each of the three non-active site cysteines to a serine (C66S, C80S and C141S). Both constructs were synthesized and inserted into a modified pET21a vector by ligation independent cloning as described [16]. Both genes were codon-optimised for expression in *Escherichia coli*. The vector includes a N-terminal His_6_-tag followed by a linker containing a recognition site for the tobacco-etch virus (TEV) protease. Design of the constructs was informed by bioinformatics analysis (HMMTOP [17, 18], and PSIPRED [19] to remove the predicted N-terminal transmembrane helix, and maximise solubility of the purified protein. Sequence verified plasmids were routinely amplified in *E*. *coli* TOP10 cells cultured at 37°C with orbital shaking (200 rpm) in LB broth supplemented with ampicillin (100 μg/mL), and subsequently isolated with a QIAprep Spin Miniprep Kit (QIAGEN).

For biochemical assays CtDsbA and CtDsbA-SSS were expressed in *Escherichia coli* BL21 (DE3) pLysS cells using ZYP-5052 autoinduction medium [20] in the presence of ampicillin (100 μg/mL) and chloramphenicol (34 ug/mL). Cultures were incubated at 30°C, for 16 h with orbital shaking at 200 rpm. Harvested cells were re-suspended in a solution of 25 mM HEPES pH 7.5, 150 mM NaCl (Buffer 1), DNAse and protease inhibitors and lysed using a constant pressure cell disrupter. Clarified lysate was purified with Talon^®^ resin (Clontech, Australia) washing with 25 mM HEPES pH 7.5, 500 mM NaCl, 2.5 mM imidazole and eluting with Buffer 1 and 500 mM imidazole. Purified protein was dialysed against Buffer 1 to remove imidazole prior to cleavage of the N-terminal His_6-_tag by treatment with a His-tagged TEV protease. TEV cleaves leaving two non-endogenous amino acids (S -1 and N 0) at the N-terminus of the protein. Contaminating TEV protease and uncleaved CtDsbA were removed by a second immobilised metal affinity chromatography step prior to a final size exclusion step in Buffer 1 using a Superdex S75 column. As required CtDsbA and CtDsbA-SSS were reduced and oxidized by 20 fold molar excess of DTT or 100 fold molar excess of oxidized glutathione, respectively. The protein redox state was confirmed by Ellman’s reagent [21].

For crystallization experiments CtDsbA was prepared as described above with the following differences. Harvested cells were re-suspended in a solution of 50 mM Tris pH 7.6, 500 mM (NH_4_)_2_SO_4_ (Buffer 2), 0.5% Triton X-100, DNAse and protease inhibitors, and lysed by sonication. Immobilized-Metal Affinity Chromatography (IMAC) purification was performed with Profinity resin (Biorad) equilibrated in Buffer 2 supplemented with 10 mM imidazole. Following wash steps (5 column volumes of Buffer 2), purified protein was eluted in Buffer 2 supplemented with 500 mM imidazole. Purified protein was dialysed against 50 mM HEPES pH 7.6, 500 mM (NH_4_)_2_SO_4_ and 0.5 mM of the reducing agent tris-(2-carboxyethyl)fosfin (TCEP) prior to TEV cleavage and a second IMAC step. Protein purity (>95%) were confirmed by SDS-PAGE analysis.

### Insulin reduction assay

DsbA proteins catalyse the reduction of the disulfide bond formed between chain A and B of insulin. When the disulfide bond is reduced, chain B of insulin precipitates. This reaction can be monitored by an increase in absorbance at 650 nm [22]. Reaction mixtures were prepared in 1 mL cuvettes with 10 μM protein, 0.33 mM DTT and 2 mM EDTA in 100 mM NaH_2_PO_4_/ Na_2_HPO_4_ pH 7.0. The reaction was started by adding insulin (IO516, Sigma Aldrich, Australia) at a final concentration of 0.13 mM. Plotted data shows mean and standard deviation (SD) for three biological replicates.

### Peptide oxidation assay

The redox activity of a disulfide bonded protein can be assessed by its ability to oxidatively fold the peptide CQQGFDGTQNSCK which has a N-terminal europium ion (Eu^III^) in an amide-coupled tetraazacyclododecane-1,4,7,10-tetraaceticacid (DOTA) chelate and a C-terminal coumarin chromophore (AnaSpec, USA) [23–25]. Upon oxidative folding the two terminal tags come in close proximity resulting in a detectable fluorescent resonance energy transfer (FRET) effect. The assay was performed in a 384-well plate (Perkin Elmer, USA). A solution of 50 mM MES, 50 mM NaCl, 2 mM EDTA, pH 5.5, 2 mM GSSG and protein concentration of 80 nM (EcDsbA) or 320 nM (CtDsbA, MtbDsbA and αWpDsbA1) were added to the wells in 25 μL aliquots. Adding 25 μL peptide to a final concentration of 25 μM started the reaction. Change in fluorescence at 340 nm was followed using a Synergy H1 Multimode plate reader (BioTek, USA). Plotted data shows mean and SD for two biological replicates.

### Scrambled-RNaseA assay

The ability of CtDsbA to isomerize disulfide bonds was evaluated by a scrambled RNaseA (ScRNaseA) assay. To generate scrambled RNaseA, disulfides were first reduced and unfolded by incubating RNaseA overnight in 50 mM Tris-HCl, pH 8 in the presence of 6 M GdmCl and 150 mM DTT at room temperature. The reduced and unfolded protein was acidified with 100 mM acetic acid/NaOH pH 4 and purified over a GE-25 Sephadex desalting resin. The eight free thiols were confirmed by Ellman’s reagent. To randomly oxidized disulfides, the reduced and unfolded RNaseA was diluted to 0.5 mg/ml in 50 mM tris-HCl, pH 8.5, 6 M GdmCl and incubated in a dark room at room temperature for at least 5 days. The randomly oxidized RNaseA (ScRNase) was concentrated, acidified and purified as described above, and oxidation of the disulfide bonds was confirmed by Ellman’s reagent. Isomerase activity was evaluated by following the renaturation of ScRNaseA spectrophotometrically. RNase A with native disulfide bonds is able to cleave cyclic-2’,3’-cytidinemonophosphate (cCMP) into 3’-cytidinemonophospate (3’CMP) resulting in an increase in absorption at 296 nm. EcDsbA, EcDsbC and CtDsbA (10 μM final concentration), were added to 100 mM sodium phosphate pH 7, 1 mM EDTA and 10 μM DTT and incubated for 5 min. To start the assay ScRNase was added at a final concentration of 40 μM. Native RNase and ScRNase (40 μM) were included as controls. At multiple time points from 0 to 360 min after initiation of the assay a 50 μL aliquot of each reaction was added to 150 μL of 3mM cCMP and the increase in absorbance at 296 nm was monitored every 10 second for 3 min. 3 biological replicates were performed for CtDsbA.

### Motility assay

In order to investigate whether CtDsbA can complement EcDsbA, the *CtdsbA* gene was cloned into the expression vector pBAD33 [26] under the control of an arabinose inducible promoter and 3’ of an *E*. *coli* specific signal peptide sequence. *EcdsbA* was likewise cloned into pBAD33 as a control. *E*. *coli* strains deficient in EcDsbA (JBC817) and EcDsbA and DsbB (JBC818) [27] were transformed with pBAD33-*CtDsbA* and pBAD33-*EcDsbA* and grown on LB agar. Liquid cultures (20 mL) were grown in M63 minimal media lacking cysteine and methionine. Each culture was normalized to an OD_600_ of 1.0. 2 μL culture containing ~10^7^ cells was used to carefully inoculate M63 minimal media soft agar in the presence or absence of arabinose (1 mg/mL). Plates were incubated at 37°C. The zone of motility was measured after approximately 7 h.

### Relative stability of oxidized and reduced forms of DsbA

The thermal unfolding of reduced and oxidized CtDsbA determined using a Jasco J-810 circular dichroism (CD) spectropolarimeter. Measurements were carried out with 10 μM protein in 100 mM NaH_2_PO_4_/Na_2_HPO_4_, 0.1 mM EDTA, pH 7.0 in a 1 mm quartz cuvette. The unfolding was monitored as a change in molar ellipticity at 220 nm with a heat rate of 0.5 K/min from 298 K to 398 K. Plotted data shows mean and SD for two biological replicates.

### Determination of the redox potential

The standard redox potential of CtDsbA was determined following a shift in electrophoretic mobility upon reduction of the active site disulfide. Samples were prepared with fully oxidized CtDsbA-SSS in degassed 100 mM sodium phosphate pH 7.0, 1 mM EDTA and 20 mM oxidized DTT and varying concentration of DTT (0 μM-16 mM) and equilibrated at 30°C. After 24 hours the reaction was stopped by adding 1/5 of the reaction volume of 10% 2,4,6-Trichloroanisole (TCA). The precipitated protein was collected by centrifugation at 13,300 g for 10 min at 4°C. The pellets were washed with 200 μL ice cold 100% acetone and dissolved in 10 μL 50 mM Tris-HCl pH 7.0, 1% SDS, 2 mM AMS. The difference in migration between the reduced and oxidized CtDsbA-SSS was determined on a 12% SDS Bis-Tris PAGE (Life Technologies, USA). The gel was stained and the intensity of the bands were analysed by ImageLab (BIO-RAD) The equilibrium constant *K*_eq_ and the redox potential E^0^ were calculated as described previously [28]. Plotted data shows mean and SD for four biological replicates.

### Determination of p*K*_a_ of Cys38 of CtDsbA

The p*K*_a_ of the nucleophilic active site cysteine, Cys38, of CtDsbA was determined by following the pH-dependent specific absorption of the thiolate anion at 240 nm. The pH-dependent absorbance at 240 nm was measured for the oxidized protein as a reference.

20 μM samples of reduced and oxidized CtDsbA were prepared in 10 mM K_2_HPO_4_, 10 mM boric acid, 10 mM sodium succinate, 200mM KCl and 1 mM EDTA over a pH range of 1.6–8.0, in a total volume of 200 μL. Absorbance at 240 nm and 280 nm was measured in a Synergy H1 multimode plate reader (Biotek, USA). The pH-dependent absorbance of the thiolate anion was corrected for, buffer absorbance, non-specific absorption at 240 nm and variations in protein concentrations and fitted to the Henderson-Hasselbalch equation.

### Crystallisation of CtDsbA

CtDsbA was crystallised using The University of Queensland Remote Operated Crystallisation X-ray Facility (UQROCX), the vapour-diffusion method and hanging drops. 96 well plates (200 nL of protein and 200 nL of reservoir solution, against 75 μL reservoir solution) were set using a Mosquito crystallisation robot (TTP Labtech) against commercially available crystallisation screens. All crystallisation experiments were maintained at 298 K. Screening of commercially available crystallisation screens with CtDsbA at a concentration of 26 mg/mL identified an initial condition (100 mM BisTris propane pH 9.0, 3.1 M potassium formate) that yielded both salt crystals as well as protein crystals. Subsequent investigation of additives was performed in 24 well plates with (2 μL protein and 2 μL crystallisation condition, against a 500 μL reservoir volume) and resulted in an optimized crystallization condition of 100 mM BisTris propane pH 9.0, 3.1 M Sodium formate and 4% Tacsimate^™^.

### X-ray data collection, structure solution and refinement

Crystals were cryocooled in liquid nitrogen without additional cryoprotectant. X-ray data were collected at 100 K at UQROCX using a Rigaku FR-E Superbright X-ray generator and a Rigaku Saturn 944 CCD area detector. Data were collected over a 360° rotation at a wavelength of 1.54187 Å. Using the autoPROC software toolbox [29] the data were indexed and integrated with XDS [30], prior to further processing with Pointless and scaling with Aimless [31]. A high resolution limit of 2.7 Å was applied to the data following evaluation of the half-dataset correlation coefficient, R_meas_ and completeness values in Aimless. As a result of this prioritisation, the I/σI value in the highest resolution shell is high (5.9). The space group was determined to be *P*2_1_2_1_2 with two copies of CtDsbA in the asymmetric unit. We note that Xtriage analysis flagged L-test anomalies that may stem from data quality deficiencies. Merohedral and pseudo-merohedral twin laws are not possible in this lattice. All phasing and model refinement procedures were implemented within the Phenix software suite. The data were phased using molecular replacement methods with AutoMR using *Bacillus subtilis* BdbD (PDB ID, 3EU3, [10]) as a search model. The search model was prepared for molecular replacement using Sculptor, with additional trimming of predicted loop regions informed by inspection of sequence alignment analysis with CtDsbA. The resulting model was subject to automated building and rebuilding using AutoBuild followed by iterative rounds of refinement (phenix.refine [32])—including refinement using hydrogen atoms using a riding model—and model building using COOT [33]. The quality of the final model was assessed with Molprobity [34] throughout the refinement process.

The electron density map supported building of an almost continuous model of residues in chain A from residue 6–203. There was insufficient density to model residues 133–135 inclusive in a loop connecting H4 and H5. Lys16 and Tyr17 on chain A are modeled as alanine as it was not possible to resolve the side chain atoms of either residue satisfactorily. Chain B extends from residues 3 to 203 with breaks from 110–111 and 133–134 inclusive in two separate loop regions. Both molecules in the asymmetric unit are structurally highly similar, and superpose with a RMSD of 1.08 Å (191 equivalent Cα atoms). Inspection of the structural alignment indicates that the main-chain atoms of all major secondary structure elements superimpose almost exactly. Structural differences between the two molecules are limited to the most N-terminal residues (residues 3–18) and several loop regions connecting: B1-B2 (residues 23–28), H2-H3 (residues 88–93), H3-H4 (residues 109–116 and H4-H5 (residues 132–136). As noted above the H4-H5 loop is incomplete in both chains, and the H3-H4 loop is incomplete in chain B. Additionally there are minor deviations in the position of the C-terminal end of H7. Notably the loops that constitute the catalytic surface—including the active site—are structurally equivalent. Further analysis of CtDsbA is restricted to chain B. All figures depict chain B.

There is additional electron density adjacent to the non-catalytic disulfide bridge (Cys 84-Cys 145) suggestive of a small molecule ligand. We attempted to identify this ligand by searching a library of 200 common ligands using the Ligand Identification module in Phenix. This relatively blind search strategy proposed succinic acid (a component of the crystallisation reagent Tacsimate^™^) as a potential ligand. Refinement of a model including succinic acid resulting in an acceptable fit to the electron density but an elevated R_free_ factor. For comparison we also modelled another potential ligand (2-oxoglutaric acid) identified by the search which we do not believe to have been present during protein purification or crystallisation, and this yielded a similar density fit and elevated R_free_ factor. User guided searching and modelling of other known crystallisation components did not yield a better fitting ligand. In light of the moderate resolution of the data and as we cannot identify a ligand with confidence, this density remains unmodelled.

Data-collection and refinement statistics are summarized in Table 1. The final refined structure has been deposited in the Protein Data Bank (PDB ID 5KBC.) All structural figures were generated using Pymol (PyMOL Molecular Graphics System, version 1.6 Schrodinger, LLC, http://pymol.org) and Adobe Illustrator.

**Table 1:** X-ray data measurement and refinement statistics for CtDsbA.

**Data collection and processing**	
Wavelength (Å)	1.54187
Resolution range (Å)	43.3–2.7 (2.8–2.7)
Space group	P 2_1_ 2_1_ 2
Unit cell dimensions	
*a*,*b*,*c* (Å)	92.2, 98.1,44.8
α, β, γ (°)	90.0, 90.0,90.0
Rmerge	0.101 (0.401)
Rmeas (within I+/I-)	0.110 (0.429)
Rmeas (all I+ & I-)	0.109 (0.432)
Total number of observations	82358 (737)
Total number unique	11646 (102)
Mean((I)/sd(I))	19.6 (5.9)
Mn(I) half-set correlation CC(1/2)	0.998 (0.880)
Completeness (%)	100 (86.4)
Multiplicity	7.1 (7.2)
**Refinement and model quality**	
**Refinement**	
^‡^R-factor (%)	24.76 (30.84)
^§^R-free (%)	28.23 (38.54)
Number of atoms	6176
macromolecules	3083
ligands	
water	22
Protein residues	390
**R.M.S.D from ideal geometry**	
RMS(bonds) (Å)	0.003
RMS(angles) (°)	0.69
**Molprobity analysis**	
Ramachandran favored (%)	96.84
Ramachandran outliers (%)	0.26
Clashscore*	6.36 ((100^th^ percentile* (N = 186, 2.706 Å ± 0.25Å))
Molprobity score**	1.74 ((100^th^ percentile* (N = 5290, 2.706 Å ± 0.25Å))
Average B-factor (Å^2^)	45.90
macromolecules	45.90
Solvent	44.20

Values in parentheses refer to the highest resolution shell

^‡^ *Rfactor* = Σ_*h*_||*F*_*obs*_|_*h*_ − |*F*_*calc*_|_*h*_ | /Σ_*h*_|*F*_*obs*_|_*h*_, where *h* defines the unique reflections

^§^ R_free_ calculated over 5.0% of total reflections excluded from refinement

* Clashscore: 100^th^ percentile is the best among structures of comparable resolution; 0^th^ percentile is the worst. For clashscore the comparative set of structures was selected in 2004, for MolProbity score in 2006

** MolProbity score combines the clashscore, rotamer, and Ramachandran evaluations into a single score, normalized to be on the same scale as X-ray resolution

## Results

### The *Chlamydia trachomatis* genome encodes a DsbA with multiple cysteine residues

*C*. *trachomatis* DsbA was first identified using a bioinformatic interrogation of all publicly available and complete prokaryotic genomes, using the Complete Microbial Resource at the Craig Venter Institute and the DsbA sequences from *E*. *coli*, *S*. *aureus* and *W*. *pipientis* as query terms [11]. This analysis returned a single gene in *C*. *trachomatis* (strain: 434/Bu, serotype: L2) predicted to encode a DsbA-like protein (Gene ID 5858475; UniProt CTL0429) and hereafter referred to as CtDsbA. We note that this gene is currently annotated as DsbG in UniProt, but based on its 15% sequence identity with EcDsbA, and 20% and 21% sequence identity with SaDsbA and *Mycobacterium tuberculosis* DsbA, respectively, we hypothesized that it is in fact a DsbA-like protein. Unlike EcDsbA or SaDsbA, but in common with MtbDsbA, *P*. *aeruginosa* DsbA2 and WpDsbA1, the predicted CtDsbA encodes additional cysteine residues outside of the active site motif. The full-length encoded CtDsbA protein has six cysteines in total. Bioinformatic analysis using multiple algorithms indicated that CtDsbA has a predicted transmembrane region from residues 11/12–28/29. From residue 29 onwards the protein sequence is predicted to face the outside of the cytoplasmic membrane (as described in Materials and Methods). We designed an expression construct restricted to the expected mature soluble CtDsbA protein starting at residue 34 to ensure no inadvertent inclusion of the predicted transmembrane helix. This construct has five cysteines.

### CtDsbA catalyzes the reduction of insulin

**Fig 1:**
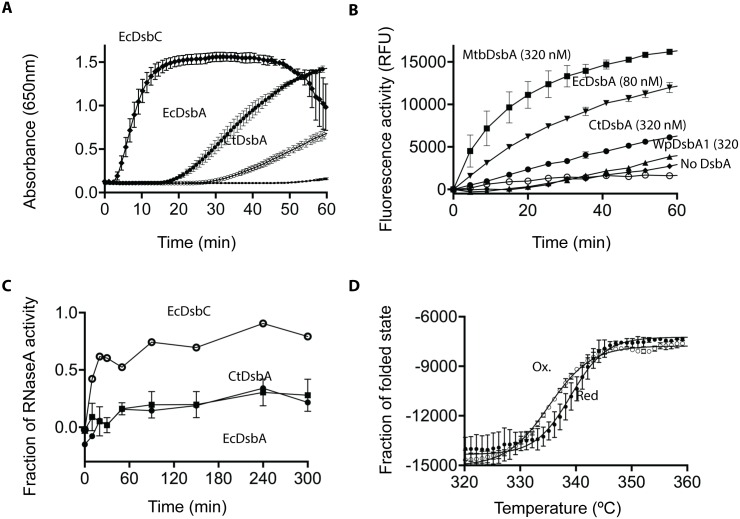
Biochemical characterization of CtDsbA. **A** Reduction of insulin (131 μM) was measured as increase in absorbance at 650nm in 0.1mM sodium phosphate buffer, pH 7, 2mM EDTA. The reaction was performed in the absence (■) or presence of 10 μM EcDsbC (●), EcDsbA (♦) or CtDsbA (○). Representative data are shown for the absence and presence of 10 μM EcDsbA. Mean and SD are shown for two biological replicates (three biological replicates for CtDsbA). **B** 80 nM EcDsbA (**▼**) and 320 nM CtDsbA (●), MtbDsbA (■) and WpDsbA1 (**▲)** oxidize a fluorescently labeled protein in the presence of 2 mM GSSG. GSSG shows only limited oxidizing activity in the absence of a DsbA protein (■). The buffer only control (○) shows no oxidizing activity. For MtbDsbA, WpDsbA1, EcDsbA and CtDSbA mean and SD of two biological replicates are shown (for each biological replicates four technical replicates was performed.) For the buffer and GSSG only controls, mean and sd for four technical replicates are shown. **C** Isomerase activity was assessed as the ability to refold scrambled RNAseA. ScRNase (40 μM) was incubated in 0.1 M sodium phosphate buffer pH 7.0, 1 mM EDTA, 10 μM DTT in the absence and presence of 10 μM EcDsbA (■), EcDsbC (○) and CtDsbA (●). RNase activity was monitored as the cleavage of cCMP which leads to an increase in absorbance at 296 nm. Mean and SD for three biological replicates is shown for CtDsbA. EcDsbC and EcDsbA is able to restore ~80% and ~20% of RNaseA activity, which is equivalent to what has been reported previously [8] **D** Temperature induced unfolding of oxidized (○) and reduced (●) CtDsbA was determined by far UV CD spectroscopy. The thermal unfolding of CtDsbA results in an increase in molar ellipticity at 220 nm showing that the reduced form of CtDsbA is more stable than the oxidized form. Mean and SD are shown for two biological replicates.

To investigate CtDsbA activity we first evaluated its redox activity in the classic insulin reduction assay. Isomerases such as EcDsbC catalyze this reaction very rapidly whereas oxidases, such as EcDsbA, catalyze the reaction much more slowly. We found that CtDsbA is able to catalyze the reduction of insulin more slowly than EcDsbA and EcDsbC (Fig 1A). The weak insulin reduction activity of CtDsbA relative to EcDsbC suggests that CtDsbA is more likely to be an oxidase than isomerase or reductase.

### CtDsbA has oxidase activity *in vitro*

The oxidase activity of CtDsbA was further investigated by its ability to oxidize a fluorescently labeled peptide substrate containing two cysteine residues. Like EcDsbA, CtDsbA was able to oxidize the model peptide substrate but a similar response required a concentration of CtDsbA four times that of EcDsbA (Fig 1B). At equivalent concentrations, the activity of CtDsbA is similar to the DsbA-IIb protein, WpDsbA1 but markedly less than that of the DsbA-IIa protein MtbDsbA. These results indicate that CtDsbA has oxidase activity in this assay, but either is intrinsically less active than EcDsbA and the class IIa protein MtbDsbA or does not interact with the substrate as avidly as EcDsbA and MtbDsbA.

### CtDsbA does not have isomerase activity in the scrambled RNase assay

To determine whether CtDsbA has disulfide isomerase activity we measured the ability of CtDsbA to isomerize the non-native disulfide bonds of scrambled RNaseA (ScRNaseA). Whereas the isomerase EcDsbC was able to restore about 80% of RNaseA activity over the course of the experiment, CtDsbA was only able to restore approximately 20% of RnaseA activity in this same time period (Fig 1C); this is comparable to the activity of the oxidase EcDsbA. Together these data indicate that CtDsbA does not have isomerase activity and behaves more like an oxidase in this assay.

### The active site disulfide in CtDsbA is destabilizing

The disulfide bond in DsbA proteins is typically destabilizing, in contrast to most proteins where disulfide bonds generally confer stability. We found that CtDsbA also has a destabilizing disulfide as the melting temperature (Tm) for the reduced form (339.1 K ± 0.2) is 4 K greater than the oxidized form (335.1K ± 0.1) (Fig 1D).

### CtDsbA is less oxidizing than *E*. *coli* DsbA

**Fig 2:**
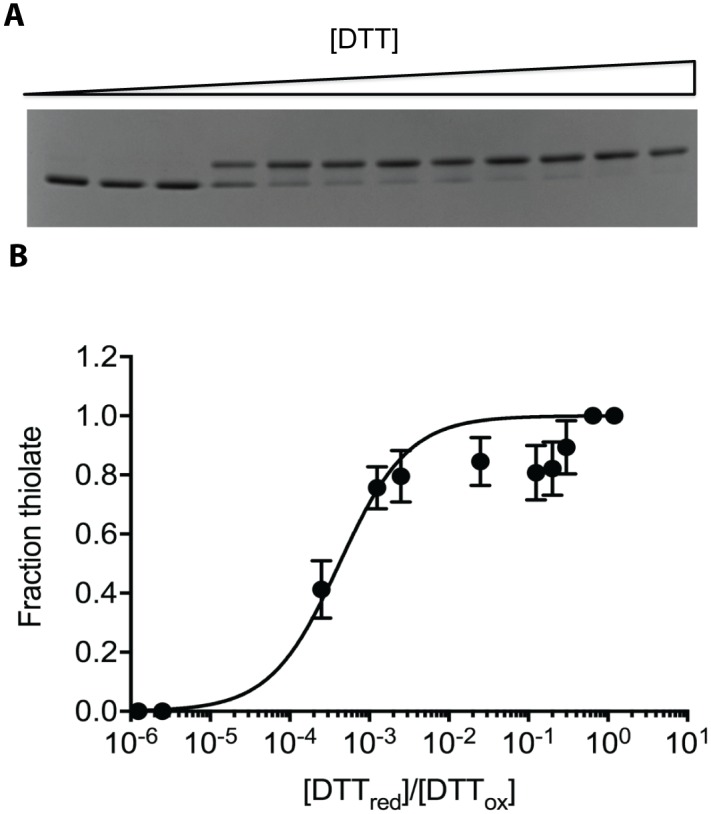
Redox potential determination for CtDsbA-SSS by electrophoretic motility shift. **A** SDS-PAGE gel of oxidized CtDsbA (3 μM) incubated for 24 h with increasing concentration of DTT (0 μM -12 mM). **B** The fraction of thiolate as a function of reduced DTT versus oxidized DTT is plotted. Fitting of the data revealed a Keq of 3.8 ± 0.8 x 10^−4^ M equivalent to a redox potential of -229 mV. Mean and SD calculated from 4 biological replicates are plotted.

The redox potential of CtDsbA in equilibrium with DTT was determined by monitoring the difference in electrophoretic mobility between the reduced and oxidized form (Fig 2). To measure the redox potential of the active site disulfide, we used the CtDsbA-SSS construct that only contains the two cysteines in the active site. We found a *K*_eq_ of 3.7 ± 0.8 × 10^−4^ M corresponding to a redox potential of -229 mV. This makes CtDsbA a much weaker oxidase than EcDsbA (E° -122 mV) and to our knowledge, the most reducing DsbA characterized to date.

### CtDsbA cannot rescue deletion of *dsbA* in *E*. *coli*

**Fig 3:**
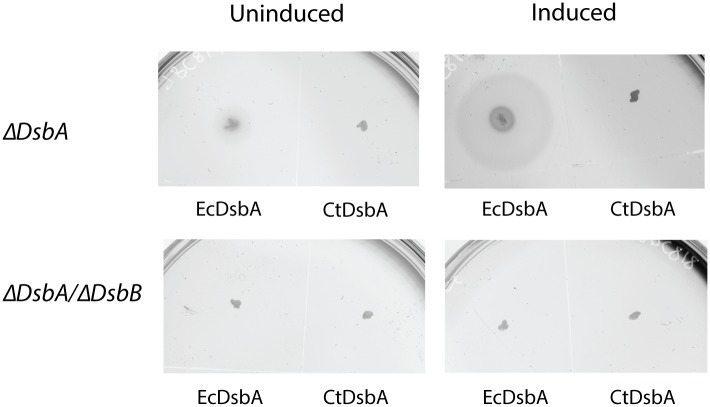
CtDsbA does not complement EcDsbA. *ecdsbA* null *E*. *coli* are unable to swarm on soft agar. Exogenous expression of EcDsbA under an arabinose inducible promoter is able to complement this non-motile phenotype as shown by the swarming halo around the original inoculation point (upper panel, EcDsbA induced). CtDsbA is not able to restore the phenotype. Neither EcDsbA nor CtDsbA are able to restore mobility in a *ecdsbA/ecdsbB* double null *E*. *coli* strain (lower panels) indicating the requirement for EcDsbB to maintain a pool of oxidized DsbA.

Deletion of the *dsbA* gene leads to pleiotropic phenotypes in *E*. *coli* [27]. One phenotype associated with DsbA-deficient *E*. *coli* is the loss of motility due to a failure to assemble functional flagella [35]. CtDsbA was expressed in Δ*dsbA* and Δ*dsbA/dsbB* backgrounds to determine if CtDsbA can complement EcDsbA. Exogenous expression of EcDsbA restored motility in Δ*dsbA E*. *coli* on soft agar (zone of motility 21 mm in comparison to 6 mm in the uninduced control; Fig 3) but as expected could not rescue the phenotype in Δ*dsbA/dsbB* bacteria. Expression of CtDsbA did not restore motility in either the Δ*dsbA or* Δ*dsbA/dsbB* bacteria, suggesting that CtDsbA cannot complement EcDsbA deficiency in *E*. *coli*.

### The crystal structure of CtDsbA features an uncommon dipeptide catalytic motif

We solved the crystal structure of CtDsbA, which was determined to 2.7 Å resolution by using molecular replacement methods. The resulting model was refined to a final R_factor_ and R_free_ of 24.8% and 28.2%, respectively. Full details of data collection and refinement parameters, and additional model quality indicators can be found in Table 1.

**Fig 4:**
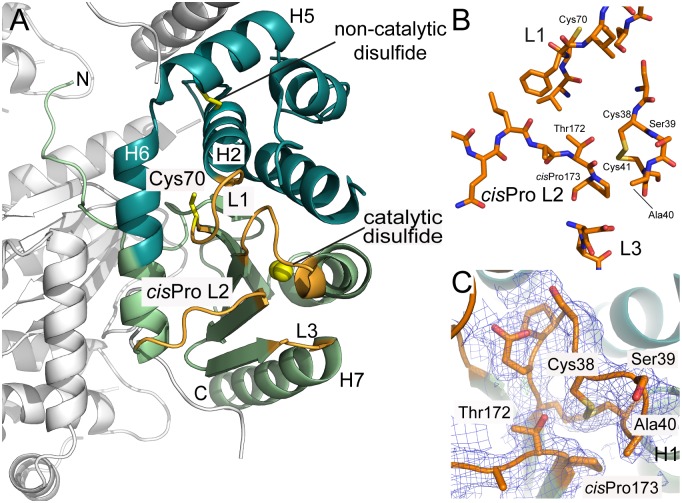
Crystal structure of CtDsbA. A. The crystal structure of CtDsbA contains a thioredoxin domain (light green) and a helical domain (dark green.) Loops on the catalytic surface that constitute the active site of CtDsbA and determine redox activity are colored orange and labeled. The active site catalytic disulfide is highlighted with sulfurs shown as yellow spheres. The non-catalytic disulfide (between Cys84 and Cys145) and the single thiol (Cys70) in L1 are shown in stick representation. The most N-terminal region of CtDsbA is unstructured. Crystal packing interactions with the second monomer in the asymmetric unit and a symmetry related molecule (shown in white) stabilize this region of the protein such that is well resolved in the electron density map. B. Close view of the four loops (L1, *cis*Pro L2, L3 and the Cys-Ser-Ala-Cys motif) which constitute the active site surface of CtDsbA. C. In the crystal structure the active site cysteines are oxidized. Analysis of bond distances indicates that the Cys 38 thiolate could be stabilized by favorable bond interactions with Thr 172 (3.4 Å between Thr 172 OH and Cys 38 SG in the oxidized structure) of the neighboring cisPro L2 consistent with an oxidizing protein character. The Cys 41 sulfur atom is 3.5 Å from the Thr 172 hydroxyl in the oxidized structure. 2Fo-Fc and Fo-Fc electron density maps for the active site and cisPro Loop 2 were generated from calculated phases using phenix.maps and are shown contoured at 1.0 σ and 3.0 σ respectively. The maps are shown within a 1 Å radius of each atom of each loop.

Consistent with all structurally characterized DsbA proteins, CtDsbA comprises a thioredoxin domain into which a second helical domain is inserted (Fig 4A). The catalytic face of DsbA proteins is composed of a canonical Cys-Xaa-Xaa-Cys motif (where Xaa is any amino acid) positioned at the N-terminal end of H1, and three additional neighboring loops: Loop 1 (linking B3 and H2), the so-called *cis*Pro Loop 2 (linking H6 and B4) and Loop 3 (linking B5 and H7) (Fig 4A). Together these loops determine DsbA enzymatic activity, modulate redox character and govern interactions with both substrate proteins and the redox partner protein DsbB [16].

In CtDsbA, the active site motif is Cys-Ser-Ala-Cys (Fig 4B). Commonly the active site motif of DsbA proteins features a Pro at the X_1_ position: 78% of DsbA proteins identified in prokaryotic genomes have a Pro at this position [11]. Furthermore the active site motif commonly includes an aromatic, polar or positively charged side chain at position X_2_ (just 13% of DsbA proteins identified in prokaryotic genomes lack such a residue at this position [11]). Via extensive mutational and biochemical analyses it has been established that the dipeptide sequence is a very important—although not the sole—determinant of thiol-disulfide oxidoreductase potential and function [36–38]. CtDsbA’s dipeptide sequence is notable in that it lacks a Pro at X_1_ and has a non-polar aliphatic residue (Ala) at X_2_. This is the first structural description of such an active site configuration. In the crystal structure Cys38 and Cys41 are oxidized, with a disulfide bond length of 2.03 Å (Fig 4B). This is unusual in that X-ray crystal structures of oxidized DsbA proteins often capture a radiation-induced reduced state (e.g. [39]). The electron density maps for CtDsbA clearly support the presence of the oxidized form (Fig 4C).

Spatially close to the active site CSAC motif is the cisPro L2 loop (3) (Ala-Thr-*cis*Pro) connecting H6 and β4. This motif (typically Xaa-Val/Thr-cisPro, where Xaa is usually an aliphatic residue) is very highly conserved among DsbA homologs and is critical for activity. In the oxidized CtDsbA structure the Cys 38 sulfur atom is 3.6 Å from the carbonyl oxygen and 3.3 Å from the side chain hydroxyl of the cisPro-1 residue Thr 172 (Fig 4C). As with other DsbA proteins, it is conceivable that in the reduced form these atoms may facilitate hydrogen bond mediated stabilization of the enzyme. We note that the Cys 38 sulfur is 3.3 Å from the side chain hydroxyl of Thr 172, which is within the range of observed atomic distances (3.1–4.6 Å) for DsbA proteins [4].

L1 and L3 play a role in partner protein interaction in other DsbA proteins. In CtDsbA L1 is 7 residues in length (Val-Cys-Phe-Ile-Arg-Gly-Ser) and is oriented towards the active site. Notably L1 contains an unpaired cysteine residue, which to our knowledge is the first structural report of an unpaired cysteine in a surface loop adjacent to the active site in DsbA (Fig 4). In EcDsbA, Loop 1 residues contribute to the binding interface with EcDsbB. In particular when Met 64, located in the middle of EcDsbA L1, is mutated to Ala the *K*_M_ of DsbA-DsbB interaction is significantly increased [40]. In CtDsbA, Cys 70 is positioned at the C-terminus of the B3 strand, and directed towards Ser 75 and Met 76 of the adjacent H2; as the side chain of Cys 70 is relatively buried in comparison to Cys 38 it is unlikely to play a role in interaction with substrate or redox partner proteins unless this loop undergoes a significant conformational change during the catalytic cycle. Finally CtDsbA Loop 3, connecting B5 and H7 is short (Asp_184_-Pro_185_-Tyr_186_) and adopts a tight turn facilitated in part by the central proline residue.

### CtDsbA has a negatively charged catalytic surface without significant pockets

**Fig 5:**
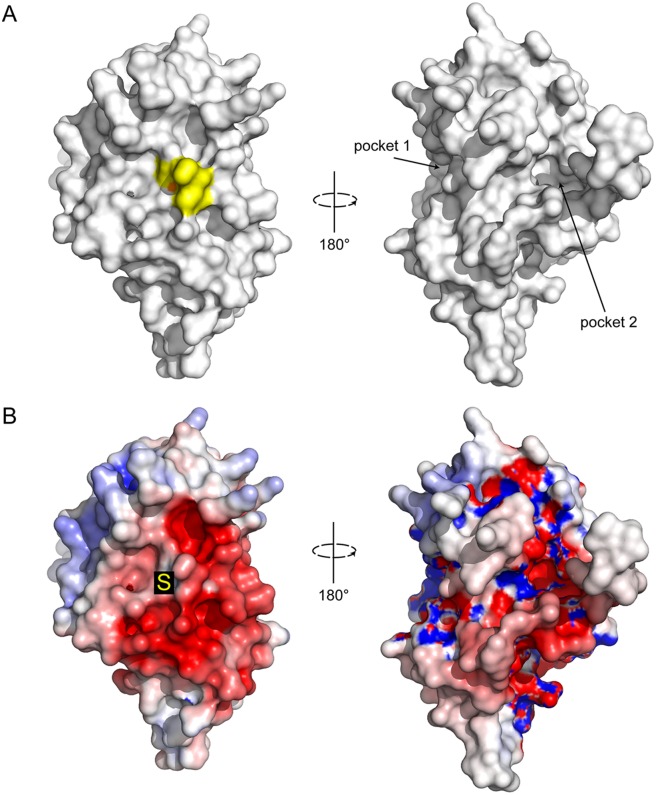
Surface properties of CtDsbA. Surface representation for CtDsbA of the catalytic (left) and non-catalytic (right) faces. The active site residues Cys-Ser-Ala-Cys are colored yellow and the nucleophilic cysteine sulfur highlighted in orange. Pockets formed on the posterior face of the protein between H1 and H3 (pocket 1) and the N-terminal unstructured region and H6 (pocket 2) are labeled. B. Electrostatic surface representation of CtDsbA. Views are oriented as above. Electrostatic surface potential is contoured between -5 (red) and +5 (blue) kT/e. The nucleophilic cysteine is annotated with an S.

Unlike EcDsbA and other *enterobacteriaciae* DsbA proteins, CtDsbA does not have a pronounced groove along its active site surface, nor are there notable pockets adjacent to the active site (Fig 5A). Small discrete pockets are present on the non-catalytic face sandwiched between H1 and H3 (pocket 1), and formed between the long N-terminal extension and H6 (pocket 2) (Fig 5A). Inspection of the electrostatic potential of the active site surface indicates it to be predominantly acidic. Of particular note is the highly acidic patch immediately adjacent to the active site cysteines on the catalytic surface (Fig 5B).

### CtDsbA has a second non-catalytic disulfide bond

**Fig 6:**
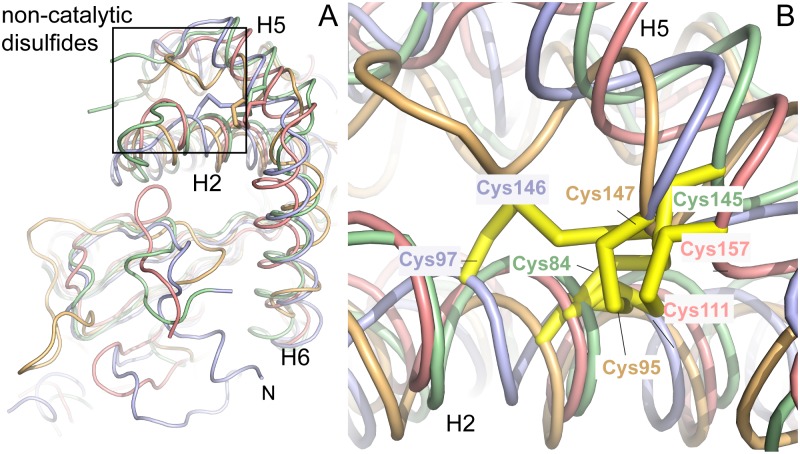
CtDsbA has a second non–catalytic disulfide bond. A. CtDsbA (green) is structurally most similar to WpDsbA1 (light blue), MtbDsbA (pink) and PaDsbA2 (orange) although it features an extended N-terminal unstructured region and lacks an additional eighth C-terminal helix present in MtbDsbA (not visible in this orientation.) Each of the four structures has a second non-catalytic disulfide bond which are very similarly positioned bracing H2 and H5 (or equivalent helices in PaDsbA2), the location of which is highlighted by a rectangular box. For clarity, depth cueing has been applied to focus on the foreground of the superimposed structures. B. Enlarged view of H2 and H5 and the non-catalytic disulfide bonds in each of the four superimposed structures. The view is oriented as in the rectangular box in panel A.

Notably CtDsbA has a second disulfide bridge formed between Cys 84 (H2) and Cys 145 (H5) stapling this helical bundle (Figs 4A and 6). A similar non-catalytic disulfide has been observed previously in the structures of WpDsbA1 [28], MtbDsbA [41] and PaDsbA2 [42]. Superposition of these four structures reveals that the position of this non-catalytic disulfide is highly similar among these four structures in each case linking equivalent helices (Fig 6).

**Fig 7:**
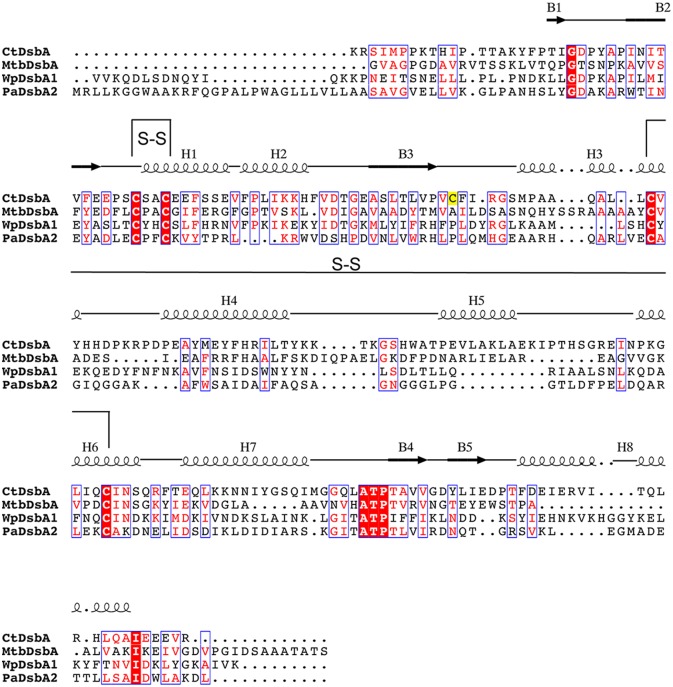
Sequence alignment of DsbAs with two disulfide bonds. Sequence alignment of structurally characterised DsbAs with two disulfide bonds. Sequences were aligned using Clustal Omega and visualized using ESPript 3.0 [53]. Secondary structural elements are shown for the structure of CtDsbA. Disulfide bonds are indicated with black connecting lines and labeled S-S. The single unpaired cysteine C70 is highlighted in yellow.

CtDsbA is the fourth structure of a DsbA protein with a second (structural) disulfide bond, permitting a detailed examination of these enzymes as a group. Overall the sequence identity of CtDsbA, WpDsbA1, PaDsbA2 and MtbDsbA is modest (15–21%; Fig 7, Table 2). The *cis*Pro Ala-Thr-Pro motif is conserved among the four proteins but the active site dipeptide is different in each and there is only limited sequence similarity in the residues immediately following the non-catalytic cysteines. Structurally they are similar overall, superimposing on CtDsbA with a RMSD of 2.5–2.6 Å (for 163–176 equivalent Cα atoms) (Table 2) although MtbDsbA is distinct at its C-terminus where it has an additional and unique eighth helix. Aside from this, structural differences are confined to loop regions. CtDsbA has a particularly extended and disordered C-terminus and two elongated loop regions: residues 128–137 linking H4 and H5, and 88–94 linking H2 and H3 relative to the other proteins. These loops are aligned in a parallel manner adjacent to one another creating a horse shape protrusion on the back of the protein, encasing a number of small pockets.

**Table 2:** CtDsbA is structurally similar to other DsbA proteins containing a second disulfide bond.

Reference protein—CtDsbA									
			Identity	TM-score	RMSD (Cα)	CXXC	L1	cisPro L2	L3
Bacterium	DsbA	PDB ID							
*C*. *trachomatis*	CtDsbA	This study	-	-	-	EEPSCSAC	VCFIRGS	ITATP	DPT
*P*. *aeruginosa*	PaDsbA2	4N30	15	0.728	2.49 (168)	ADLECPFC	NMHG	ITATP	MAD
*W*. *pipientis*	WpDsbA1	3F4R	20	0.773	2.57 (176)	ASLTCYHC	FPLDY	ITATP	GGYK
*M*. *tuberculosis*	MtbDsbA	4K6X	21	0.699	2.63 (163)	EDFLCPAC	AILDSA	VHATP	IFNNG
*E*. *coli*	EcDsbA	1FVK	15	0.598	3.45 (153)	FSFFCPHC	NFMGGC	LRGVP	NPQGMDTSN

Structural comparison of CtDsbA to three other DsbA proteins that also contain a second disulfide bond (PaDsbA2, WpDsbA1 and MtbDsbA) and the canonical single disulfide containing EcDsbA. Structures were aligned to the reference CtDsbA protein using TM-align. The resulting template modelling score (TM-score) and root mean squared deviation (RMSD, for which the equivalent number of Cα atoms involved in the structural alignment is given in parenthesis) are tabulated. TM-score is a quantitative measure of similarity between two proteins independent of their length (a TM-score >0.5 generally corresponds to the same fold in SCOP; a score closer to 1.0 implies that they are highly similar.) Sequences of the catalytic motifs and loops on the catalytic face of each protein are also detailed. Note that the sequences of the highly variable L3 loop are not aligned.

### Classification of CtDsbA

DsbA proteins have previously been segregated into distinct structural classes [4]. Assignment to one of the two major classes (DsbA-I and DsbA-II) is defined primarily by the β-sheet topology of the thioredoxin domain [4]. In the thioredoxin domain of CtDsbA, β1 hydrogen bonds to β3 to give a β-sheet topology of 1-3-2-4-5, assigning CtDsbA to the DsbA-II enzyme class. Previously identified members of DsbA-II include DsbA from the Gram positive species *S*. *aureus* and *B*. *subtilis*, *M*. *tuberculosis* and the endosymbiont Gram negative species *W*. *pipientis*. Consistent with its assignment to DsbA-II, interrogation of the Protein Data Bank using DaliLite [43] identified that CtDsbA is structurally similar to the DsbA-II proteins *B*. *subtilis* BdbD (PDB ID 3EU3, 1.9 Å rmsd, 28% sequence identity) and *W*. *pipientis* DsbA1 (PDB ID 3F4R, 2.3 Å rmsd, 20% sequence identity) as well as PaDsbA2 (PDB ID 4N30, 2.6 Å rmsd, 15% sequence identity) which although not previously classified also shares the β-sheet topology of other DsbA-II proteins and a second non-catalytic disulfide bond observed in 3 of the 4 previously assigned DsbA-II proteins (Fig 4, Table 2).

## Discussion

In the present study we demonstrate that the *C*. *trachomatis* encodes a DsbA protein (Gene ID 5858475 (UniProt CTL0429), which has oxidase activity and the structural features of a DsbA-II type protein. The weak activity of CtDsbA in the insulin reduction assay relative to EcDsbC, suggests that CtDsbA is a thiol disulfide oxidase and not an isomerase. Consistent with this, CtDsbA is able to oxidize the folding of a model peptide substrate *in vitro*, albeit less efficiently than the canonical oxidase EcDsbA. Our finding that CtDsbA is significantly less active than the isomerase EcDsbC in the ScRNase assay further supports the assertion that CtDsbA is an oxidase without disulfide isomerase activity.

Heterologous complementation studies shows that CtDsbA is not able to complement *EcDsbA* deficient *E*. *coli*. However this is not unprecedented among DsbA proteins. The DsbA-II proteins MtbDsbA and WpDsbA1, as well as the DsbA-Ib protein *N*. *meningitis* DsbA3 (NmDsbA3) are all DsbA proteins that are not able to complement EcDsbA deficiency. In common with CtDsbA these proteins are relatively structurally distinct from EcDsbA (RMSD between 2.76 and 3.71 Å, over 152–175 Cα atoms). Successful rescue of function relies upon an ability of the exogenous DsbA to interact with both the substrate flagella proteins and EcDsbB. The observed structural diversity may preclude one or both interactions and explain why they are not able to rescue DsbA activity in *dsbA* deficient *E*. *coli*.

Whilst DsbAs can be broadly very similar in their structure and function, the redox character of individual enzymes can vary considerably [16]. Like other DsbA proteins CtDsbA active site has a destabilizing disulfide bond that lowers the melting temperature of the oxidized protein by 4 K relative to the reduced state. Tm differences between oxidized and reduced DsbAs range from 1 (SaDsbA) to 15 K (NmDsbA1) (Table 3). This puts CtDsbA at the lower end of the range of a destabilizing disulfide placing it between SaDsbA, which, with a ΔTm of 1 K has a melting temperature that is barely affected by oxidation [3], and WpDsbA1 with a ΔTm of 6 K. Furthermore it suggests that the destabilizing effect of oxidation in CtDsbA is relatively mild compared to that of the enterobacterial DsbAs (ΔTm of 8–12 K for EcDsbA, SeDsbA and KpDsbA) or NmDsbA1 (ΔTm 15 K). The redox potential of CtDsbA is -229 mV. This is substantially less oxidizing than that of EcDsbA (-122 mV) and is to our knowledge, the most reducing redox potential reported for a DsbA protein. It is most similar to the redox potential of WpDsbA1 (-163 mV), and approaches that of the reducing enzyme thioredoxin (-270 mV, [44, 45])

**Table 3:** Comparison of melting temperatures, p*K*_a_ and redox potential of different DsbA proteins.

	T_m_ K) reduced	T_m_ (K) oxidized	p*K*a	*K*_*eq*_ (M)	E° (mV)
EcDsbA [2, 7]	350.9 ± 0.2	341.7 ± 0.2	3.3 ± 0.09	8.1 ± 0.2 × 10^−5^	-122
SeDsbA [16]	351.2 ± 0.2	342.8 ± 0.4	3.3 ± 0.06	12.8 ± 0.3 × 10^−5^	-126
KpDsbA [9]	347.1 ± 0.2	335.8 ± 0.3	3.2	6.14 ± 0.1 × 10^−5^	-116
VcDsbA [5],[46]	357 ± 0.2	346 ± 0.2	5.1	7.7 ± 0.03 × 10^−5^	-116
NmDsbA1 [6]	348 ± 2	333 ± 2	3.0	3.7 × 10^−6^	-79
SaDsbA [8]	345.1 ± 0.08	345.6 ± 0.09	3.4 ± 0.07	2.09 ± 0.27 × 10^−4^	-131
MtbDsbA [41]	ND	ND	4.2 ± 0.2	17.37 ± 0.1 × 10^−6^	-99
WpDsbA [28]	337 ± 0.05	331 ± 0.05	4.7 ± 0.08	2.2 ± 0.27 × 10^−3^	-163
CtDsbA	339 ± 0.2	335 ± 0.1	ND	3.7 ± 0.8 × 10^−4^	-229

Errors are provided were available. ND Not determined

The reactivity of DsbA proteins is greatly influenced by the p*K*_a_ of the nucleophilic active site cysteine. In reduced EcDsbA the thiol group of the surface exposed Cys 30 has a p*K*_a_ of 3.3 [2] which is notably lower than the typical value for a cysteine residue (~8.5). Preliminary experiments indicate that the pK_a_ of the nucleophilic cysteine in CtDsbA is slightly higher than that of EcDsbA. This observation is based on a single experiment (described in Methods) that could not be reliably repeated due to the instability of oxidized CtDsbA below pH 7. Thus the p*K*_a_ of the nucleophilic active site cysteine Cys38 in CtDsbA has not been determined. The interatomic distances suggests that the thiolate anion is somewhat stabilized by hydrogen bond interactions with the Cys41 amide N (2.8 Å in the oxidized structure) and the active site dipeptide X_2_ Ala40 amide N (3.6 Å distance in the oxidized structure, Fig 4C) as is observed in other DsbA proteins (summarized in [4]). However, the primary factor stabilizing the nucleophilic thiolate in dithiol oxidoreductases is the N-helix dipole of H1 [36, 47], which is very sensitive to the microenvironment of the helix terminus.

Notably an uncharged aliphatic Ala at position X_2_ of the active site dipeptide in CtDsbA would not destabilize the N-helix dipole as per His 32 in EcDsbA [47]. Nor can it offer direct electrostatic stabilization of the thiolate anion as per a basic [48] or aromatic amino acid (via a sulfur-aromatic ring interaction [38] at this position). Similarly the lack of a helix breaking proline in the active site dipeptide removes enhancement of the H1 dipole proposed to occur in other more oxidizing DsbA proteins [38, 49]. Together the apparent lack of factors in CtDsbA to destabilize the oxidized form of the protein, or conversely to stabilize the nucleophilic thiolate in the reduced state, is consistent with both the relatively small difference in Tm between oxidized and reduced CtDsbA, and the less oxidizing redox potential relative to EcDsbA and other CPHC dipeptide containing DsbA proteins.

CtDsbA, WpDsbA1, PaDsbA2 and MtbDsbA all have a non-catalytic disulfide in the helical domain, in addition to the active site catalytic disulfide [28, 41, 42]. The functional consequence of the second disulfide bond in CtDsbA is unknown but in WpDsbA1 it may play a regulatory role by autoinhibiting its reoxidation by WpDsbB (25). In PaDsbA2 it is reported to influence the redox potential of the active site cysteines [42] as mutation of the second disulfide results in reduction of redox potential from -67 mV to -118 mV [42]. However, this is not the case in WpDsbA1 where deletion of the additional cysteines has a very modest effect on redox potential (-170 mV for wild-type and -163 mV for the mutant [28]), although it is associated with a small increase in the p*K*_a_ (p*K*_a_ of 4.7 for the wild-type and 5.0 for the mutant) of the nucleophilic cysteine [28]. MtbDsbA and wild-type PaDsbA2 have strongly oxidizing redox potentials (-99 mV and -67 mV respectively) whereas WpDsbA1 and CtDsbA are the two most reducing DsbA proteins characterized to date. Together this suggests that the second disulfide is not a significant modifier of redox potential for enzymes containing two disulfides. Instead it is more likely that differences in redox character are a composite of contributing factors including the nature of the dipeptide sequence in the catalytic motif.

In the present study, we used a recombinant expression construct with five cysteines (one unpaired). In the full-length gene there is an additional cysteine in a predicted transmembrane region. In light of the predicted location of the additional cysteine, and the relative inaccessibility of the unpaired Cys 70 it appears unlikely that the N-terminal transmembrane cysteine and Cys 70 would interact with one another in the native protein. The high cysteine content is notable relative to other DsbAs, and may be a feature of chlamydial DSB proteins more generally; *Chlamydia pneumoniae* DsbH, a reducing dithiol oxidoreductase with structural similarity to thioredoxin and EcDsbDγ has seven cysteine residues, of which only two are engaged in a disulfide bond [13] Interestingly the chlamydial proteome has striking cysteine and disulfide features more generally; for example, during its unique developmental cycle the bacteria adopt an extracellular infectious form called an elementary body which is encapsulated by a highly complex envelope of disulfide cross linked proteins [50]. Upon infection of a host cell, these disulfide bonded proteins are reduced and the elementary body differentiates into reticulate bodies [50]. Additionally a greater proportion of secreted proteins in *C*. *pneumoniae* have an unpaired number of cysteines (57%) compared to that of *E*. *coli* (39%*)*. This has led to a hypothesis that chlamydial species may maintain a particularly reducing periplasmic environment in order to prevent misfolding of cysteine rich proteins. If so, this may be supported in *C*. *trachomatis* by the relatively low redox potential of CtDsbA.

Based on its topology we assign CtDsbA to the DsbA-II class of enzymes. DsbA-II type enzymes can be further separated into two sub-divisions (DsbA-IIa and DsbA-IIb) on the basis of catalytic surface loop configurations and surface features. The archetypal DsbA-IIa protein is MtbDsbA in which Loop 1 is oriented towards the active site motif and the protein surface is decorated with negative potential [4]. Until now WpDsbA1 has been the single representative member of the DsbA-IIb subdivision distinguished primarily from DsbA-IIa by its strikingly positive electrostatic potential around the active site, and Gram negative origin. Notably the L1 configuration in DsbA-IIb also points towards the active site; and the same conformation is adopted in CtDsbA. Similar to currently identified DsbA-II proteins the surface of CtDsbA lacks the canonical grooves observed in DsbA-I (either along the catalytic surface (DsbA-Ia) or the posterior face of the protein (more typical of DsbA-Ib.) CtDsbA is strikingly charged near its active site with a pronounced patch of negative potential in this region. Taken together the structural features of CtDsbA and similarity to other DsbA-II proteins do not definitively assign CtDsbA to one of the two DsbA-II subclasses: whilst structurally more similar overall to DsbA-IIa’s MtbDsbA with a similarly negatively charged catalytic surface, its Gram negative origin and more reducing redox potential is more consistent with that of WpDsbA1 and DsbA-IIb. It is possible that DsbA-II proteins represent a broader continuum of structural features not yet fully captured by the current repertoire of structures. We await further population of the DsbA-II class before definitively sub-categorizing CtDsbA.

In conclusion, we have demonstrated that *C*. *trachomatis* encodes a DsbA protein with oxidase activity. The structure of CtDsbA yields new insight into the protein’s relatively negative redox potential and our observation that the oxidized form of CtDsbA is only mildly destabilized relative to the reduced form. This characterization of CtDsbA expands our understanding of the range of redox activities exhibited by DsbAs. Further it is a significant addition to a growing structural library of DsbA proteins, supporting ongoing exploration of the potential for development of narrow or broad spectrum antimicrobials against this important family of virulence-associated proteins [51],[52],[9],[4].
